# The exploration of Hashimoto's Thyroiditis related miscarriage for better treatment modalities

**DOI:** 10.7150/ijms.48128

**Published:** 2020-08-29

**Authors:** Yu Min, Xing Wang, Hang Chen, Guobing Yin

**Affiliations:** Department of Breast and Thyroid Surgery, The Second Affiliated Hospital of Chongqing Medical University, No.74, Linjiang Rd, Yuzhong Dist, Chongqing 404100, China.

**Keywords:** Hashimoto's thyroiditis, autoimmune disease, miscarriage, immune system, intravenous immunoglobulin, levothyroxine

## Abstract

Hashimoto's thyroiditis (HT) is the most prevalent autoimmune thyroid disease (ATD) worldwide and is strongly associated with miscarriage and even recurrent miscarriage (RM). Moreover, with a deepening understanding, emerging evidence has shown that immune dysfunctions caused by HT conditions, including imbalanced subsets of CD4+ T-helper cells, B regulatory (Breg) cells, high expression levels of CD56dim natural killer (NK) cells, and cytokines, possibly play an important role in impairing maternal tolerance to the fetus. In recent years, unprecedented progress has been made in recognizing the specific changes in immune cells and molecules in patients with HT, which will be helpful in exploring the mechanism of HT-related miscarriage. Based on these findings, research investigating some potentially more effective treatments, such as selenium (Se), vitamin D3, and intravenous immunoglobulin (IVIG), has been well developed over the past few years. In this review, we highlight some of the latest advances in the possible immunological pathogenesis of HT-related miscarriage and focus on the efficacies of treatments that have been widely introduced to clinical trials or practice described in the most recent literature.

## Introduction

Hashimoto's thyroiditis (HT), which is also known as chronic lymphocytic thyroiditis (CLT), is currently the most prevalent autoimmune thyroid disease (ATD) 1. However, the etiopathogenesis is still incompletely defined. HT causes chronic inflammation of the thyroid tissues, and hypothyroidism in approximately 20-30% of patients, especially in the female population 2-4. Previous research has shown that HT is an independent risk factor for the occurrence and development of papillary thyroid carcinoma (PTC). Additionally, the complications of HT have a greater long-term negative impact on pregnant women.

Currently, miscarriage occurs in 8-15% of clinically recognized pregnancies and approximately 30% of all pregnancies in recent epidemiological investigations 5, 6, and chromosomal abnormalities account for approximately 50% of fetal losses in the first 8-15 weeks of gestation 7. However, while the miscarriage rate has slightly decreased in recent years 5, 6, unexplained miscarriage and even recurrent miscarriage (RM), which is defined as two consecutive spontaneous losses or three or more spontaneous losses, severely impact the physical and mental health of the female population 8. With a deepening understanding of the maternal and fetal immune response, unexplained miscarriage is closely related to an abnormally activated maternal immune system.

Although HT is regarded as one of organ-specific autoimmune diseases (ADs), numerous clinical studies have confirmed that similar to other ADs, HT plays a vital role in women's miscarriage and even RM 9-13. With the development of cellular and molecular immunology, accumulating studies have found that not only hypothyroidism but also immune system disorders 14 caused by HT conditions are involved in the pathogenesis of adverse pregnancy outcomes 15-18. During a healthy pregnancy, the upregulation of regulatory T (Treg) cells and T-helper type 2 (Th2) cells represent a major maternal adaptation that helps with embryo implantation and the maintenance of pregnancy 19-21. However, under HT conditions, the expression levels of proinflammatory cytokines are upregulated, while anti-inflammatory factors are downregulated 15, 17. Due to the complex mechanisms underlying HT-related miscarriage, which are still under exploration, especially the pathway involved in the HT-mediated abnormally activated immune response, treatment decisions mainly focus on avoiding hypothyroidism and rebalancing the function of the immune system. However, the efficacies of L-thyroxine (L-T4) substitution and other immunomodulatory drugs are inconsistent in recent research 10, 22-25.

The purpose of this review is to summarize the specific changes in immune cells and cytokines under HT conditions, which can provide a basis for the investigation of more novel immunotherapies for women with HT-related miscarriage. In addition, we discuss the efficacies of treatments that have been widely introduced to clinical practice described in the most recent literature.

## HT and miscarriage

**Figure 1:**
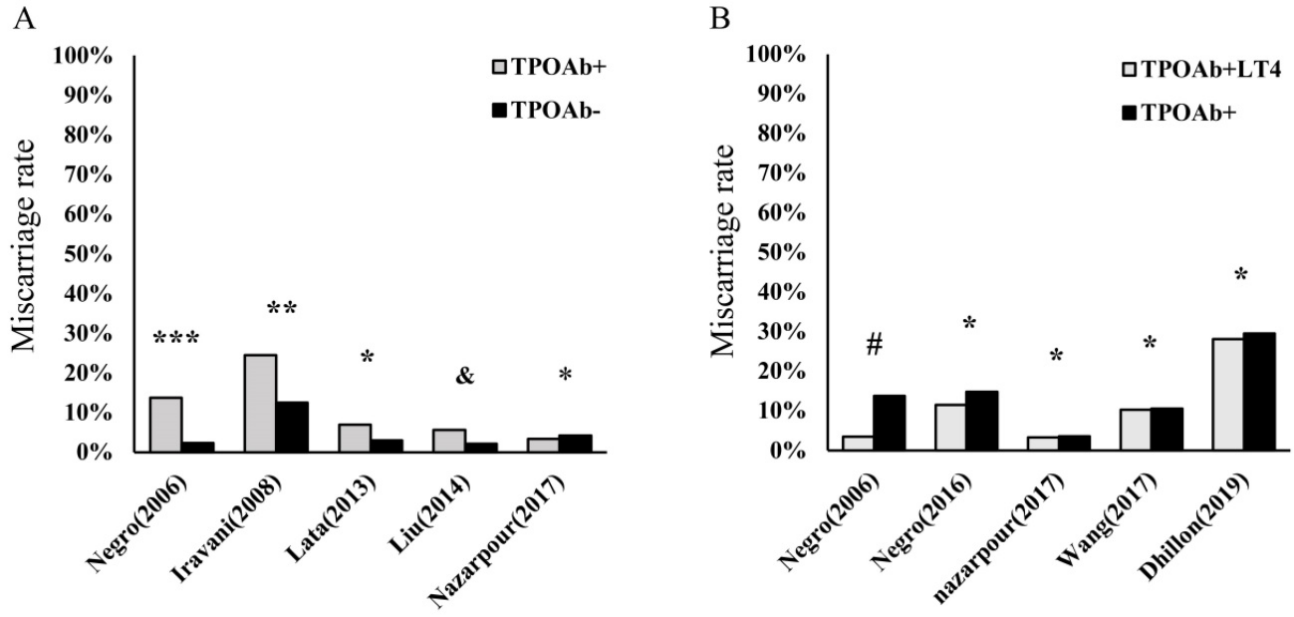
Hashimoto's thyroiditis (HT) is an important risk factor in miscarriage and even recurrent miscarriage (RM). However, the efficacies of L-T4 supplementation are still debatable. **(A).** The percentage of miscarriage in thyroid peroxidase antibody-positive (TPOAb+) pregnancies euthyroid women and health controls (TPOAb-); ^***^p< 0.0001 and ^**^p< 0.001 compared to control groups; ^*^p = no statistical significance (NS) and ^&^p= 0.002 compared to controls 11, 12, 24, 185, 186;** (B).** The percentage of miscarriage among euthyroid women with TPOAb+ who received levothyroxine treatment (TPOAb+LT4) and had no treatment or placebo (TPOAb+); ^#^*p*< 0.01 and ^*^p = NS compared to controls 10, 22, 24, 25, 185.

Currently, HT is the most prevalent organ-specific AD, with an insidious onset, and can ultimately cause hypothyroidism in nearly 20%-30% of patients 1, 2. The histological characteristics of HT include atrophy of follicular cells, diffuse lymphocyte infiltration in thyroid tissue, goiter, and fibrosis. In a recent large-scale epidemiological survey involving 78,470 participants in China 26, the prevalence of positive thyroid globulin antibody (TgAb) and thyroid peroxidase antibody (TPOAb) were 9.7% and 10.19%, respectively, with a dominance of females. Thus, HT is gradually becoming a noticeable problem in women's health, including pregnancy in women of childbearing age 11, 27, 28. Maternal hypothyroidism has been shown to be significantly associated with miscarriage, preterm birth, and growth restriction. In addition, this condition can affect hormonal changes, resulting in decreased plasma concentrations of both total testosterone and estradiol (E2) 29, 30. Consequently, L-T4 supplementation is the most important initial treatment among patients with HT to avoid overt hypothyroidism, which may help reduce the prevalence of adverse pregnancy outcomes 30-32. In contrast, numerous women with HT have been observed to be in SH, which is the precursor state to hypothyroidism. Similar to patients with overt hypothyroidism, these euthyroid patients with HT have higher rates of miscarriage and even RM than healthy control (**Figure 1A**). However, the efficacy of L-T4 supplementation among euthyroid women applied to prevent miscarriage and preterm birth induced by HT in recent research is inconsistent (**Figure 1B**). Notably, with the extended exploration of HT in recent years, an abnormally activated proinflammatory state of the immune system is found in these patients 14, 17, 18, 33-35. Therefore, these findings enrich our knowledge of HT-related miscarriage, especially in euthyroid women.

## Immune system disorders

**Figure 2:**
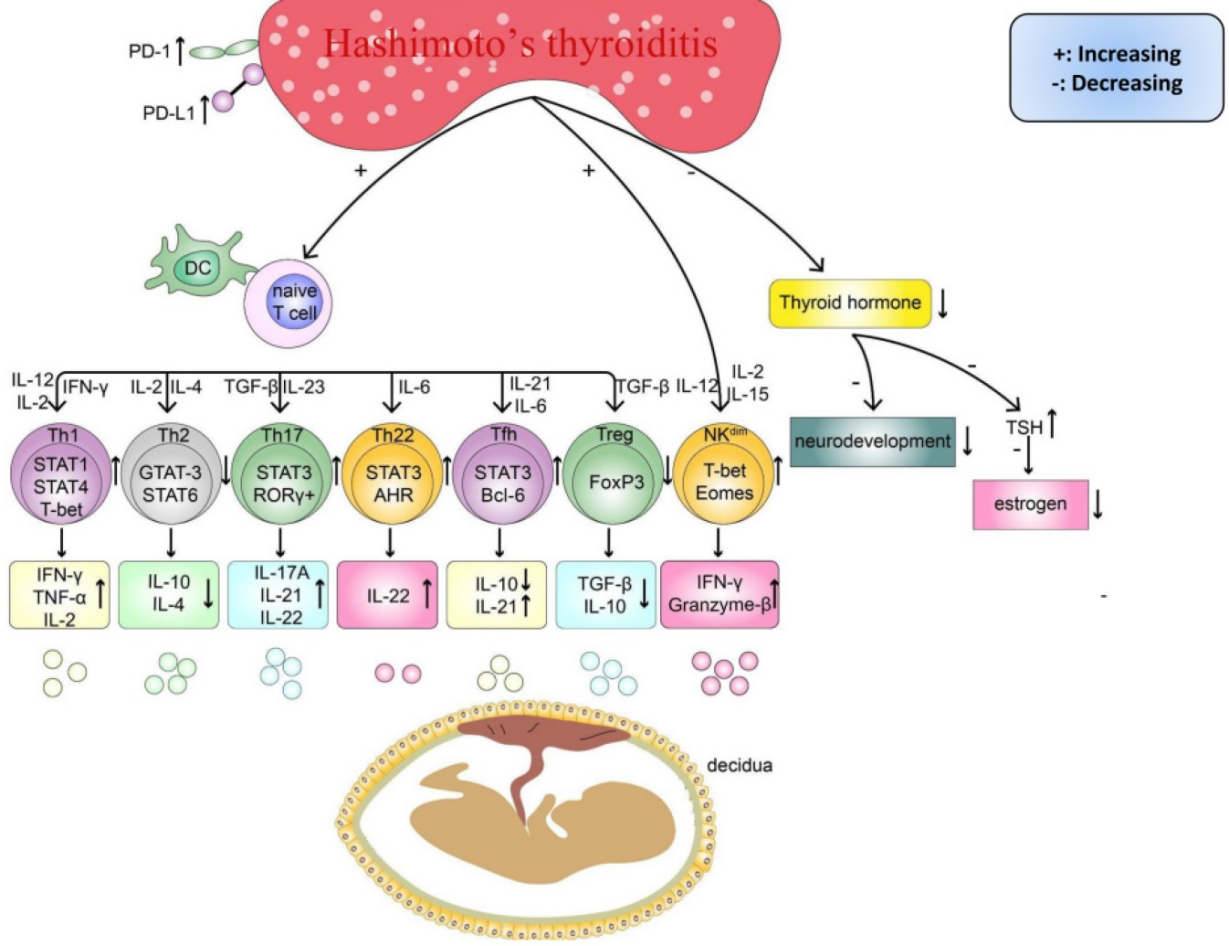
There are existing endocrine and immune disorders in HT women, which can impair pregnancy outcomes. Firstly, the imbalanced differentiation of naïve CD4^+^T cells, such as the abnormal increased Th cells (Th1, Th17, Th22, Tfh) and cytokines (IL-2, IL-6, IL-12, IL-15, IL-17A, IL-21, IL-22, IL-23, IFN-γ, TNF-α), and decreased Th cells (Th2, Treg) and cytokines(IL-4, IL-10, TGF-β). Secondly, abnormal activated NK^dim^ cell and its cytokines (IFN-γ, Granzyme B). Finally, the insufficient thyroid hormone caused by chronic inflammatory damage in the thyroid can delay the fetus's neurodevelopment and decrease the level of estrogen.

As women increasingly exhibit unexplained reproduction failure 5, 36, 37, especially under HT conditions 9-12, many researchers have attempted to reveal the possible underlying mechanisms and facilitate the development of new and better treatment approaches, particularly for subpopulations of women with RM. Previous research has found that women with thyroid autoimmunity had a higher prevalence of the copresence of non-organ-specific autoantibodies, which could add to the risk of adverse pregnancy outcomes 38, 39. Similarly, a recently published study reported a higher prevalence of RM in women with HT and nonendocrine autoimmune disorders (NEADs), such as atrophic gastritis and connective tissue diseases, than women with isolated HT (*p*<0.0001) 40. Therefore, the phenomenon of a higher risk of miscarriage in women with HT and concurrent NEADs is helpful for understanding the pathogenesis of HT-related miscarriage and suggests that patients can simultaneously benefit from effective treatments for NEADs before conceiving. Regarding HT, although the circulating TPOAb or TgAb level has repeatedly been reported to be an independent risk factor determining miscarriage, the association between different titers of these antibodies and the risk ratio of miscarriage is still debatable 40-43. Indeed, importantly, immune system disorders, including the upregulation of proinflammatory cells and downregulation of anti-inflammatory cells, are observed in the peripheral blood (PB) of patients with HT (**Table 1**). Thus, anti-thyroid antibodies are more likely markers of a potentially wider autoimmune imbalance. Additionally, some important cytokines, such as interleukin (IL)-10 and transforming growth factor-beta (TGF-β), are expressed at lower levels in maternal-fetal immune tolerance than in pregnant women without HT. Some researchers suggest that similar to other immune diseases, HT could share similar mechanisms in inducing pregnancy loss 16, 18, 44. The circulating TPOAb level 45, 46, abnormal immune system activation, an imbalance in subsets of T cells and Breg cells and a strong cytotoxic effect of natural killer (NK) cells 17, 18, 47 are possible mechanisms underlying the progression of HT-related miscarriage (**Figure 2**).

### CD4^+^ T-helper cells

Similar to other ADs, HT is often accompanied by immune dysfunction, especially imbalanced subsets of CD4^+^ T-helper cells. Consequently, the unregulated expression of cytokines secreted by different T-helper cell subsets may contribute to the progression of HT and its complications. For instance, numerous studies revealed higher levels of T-helper type 1 (Th1) and T-helper type 17 (Th17) cells but lower Th2 and Treg cells in the PB of women with HT 44, 48.

For successful implantation and fetal development during pregnancy, the proportions of naïve CD4^+^ T-helper cell subsets, which are mainly differentiated into Th1 cells, Th2 cells, Th17 cells, and Treg cells, are significantly correlated with immune tolerance at the maternal-fetal interface 19, 48, 49. On the one hand, cytokines, including interferon-gamma (INF-γ), tumor necrosis factor-alpha (TNF-α), and IL-17, secreted by Th1 and Th17 cells 50-52 can promote trophoblast differentiation and fusion during placenta formation. In addition, controlled, mild inflammation on the maternal-fetal interface and extravillous trophoblast cells (EVTs) play a crucial role in proper remodeling and invasion of maternal spiral arteries in the uterine decidua 52, 53. On the other hand, during pregnancy, cytokines, including TGF-β, IL-4, and IL-10, secreted by Th2 and Treg cells can regulate maternal-fetal immune tolerance for better embryo implantation and development 54-58. Moreover, the balance of CD4^+^ T cell subsets, especially the Th1/Th2 ratio on the maternal-fetal interface, is closely related to reproductive success 16, 59. Therefore, continuous high expression levels of proinflammatory cytokines and low expression levels of anti-inflammatory cytokines in T cell subsets in PB 60-62 can weaken immune tolerance at the maternal-fetal interface and the progression of thyroiditis (**Figure 2**). Regarding euthyroid patients with HT with (or without) NEADs, notably, an inspiring study by Santaguida et al. 63 measured the intracellular Th1 and Th2 distinctive cytokine levels (IL-2 in Th1 and IL-4 in Th2) in these patients. Correspondingly, a significant increase in Th1 cells was observed in these patients with NEADs, which was consistent with the changes in Th1 in patients with mild or severe hypothyroidism with HT 58. However, the main difference was observed in the percentages of Th2 cells between the patients with isolated HT and patients with NEADs. As these two studies discovered, a reduced percentage of Th2 cells was detected in patients with HT, and this percentage was significantly positively correlated with the disease severity 58, 63. In contrast, most patients with concurrent NEADs had a significantly increased percentage of Th2 cells and a lower Th1/Th2 ratio than the patients with isolated HT (1.78 vs. 3.8). Thus, detecting the Th1/Th2 ratio in women with HT before and during pregnancy is warranted to discover potential systemic ADs and predict the risk of adverse pregnancy outcomes; however, further research is needed to prove this possible relationship.

### B regulatory cells

**Figure 3:**
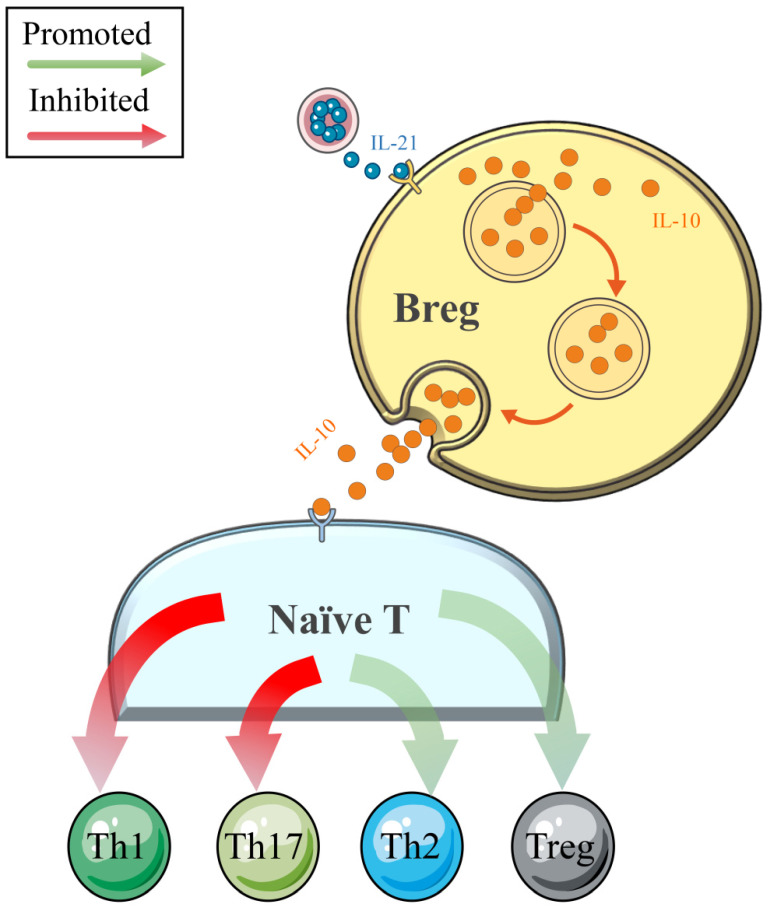
In healthy individuals, Breg cells play an important role in maintaining the crucial balance between the pool of Tregs/Th1/Th2/Th17 populations by the release of IL-10. The negative regulatory effects of Breg cells thereby limit inflammatory responses and subsequent tissue damage.

Traditionally, B cells are involved in the pathogenesis of ADs (such as systemic lupus erythematosus, SLE) through antigen (Ag)-specific autoantibody production 64. However, with the deepening of clinical and basic research, the negative regulatory effects on cellular immune responses and inflammation have been determined in Breg cells, which constitute a small subset of B cells (**Figure 3**). Numerous cytokines produced by Breg cell subsets have been determined, and IL-10 is the most widely studied 65-68. A recently published comprehensive review noted that IL-10 secreted by Breg cells could independently not only suppress Th1 and enhance Th2 polarization but also inhibit IFN-γ and TNF-α responses *in vitro* 69. However, this effect was weakened in patients with NEADs 68, 70. In a study conducted by Flores-Borja et al. 68, the efficacies of CD19^+^CD24^hi^CD38^hi^ Breg cells in the polarization of CD4^+^ T-helper cells were compared between patients with active rheumatoid arthritis (RA) and healthy controls. As the authors determined, in the healthy patients, CD19^+^CD24^hi^CD38^hi^ Breg cells could inhibit naïve T cell differentiation into Th1 and Th17 cells and convert CD4^+^CD25^-^ T cells into regulatory T cells (Tregs) by the production of IL-10. However, the number and function of CD19^+^CD24^hi^CD38^hi^ Breg cells were reduced and impaired in the patients with active RA, respectively, such that these cells could only maintain the capacity to inhibit Th1 cell differentiation 68. The phenomenon of increased expression levels but impaired negative-regulation functions of Breg cells was also observed in patients with HT 65, 66. Notably, Santaguida et al. 65 found a similar percentage of unstimulated Breg and Breg memory cells in patients with HT and healthy controls, while euthyroid patients with HT showed an increased proportion of functional Breg cells (CD19^+^CD24^hi^CD38^hi^IL-10^+^Breg cells). Moreover, these authors also demonstrated an increased number of Breg cells with reduced functional parts in patients with HT and concurrent NEADs compared with patients with isolated HT. This insightful discovery and their latest research concerning this topic 40 suggest that more attention should be paid to HT women with NEADs because these women may have more frequent and severe immune system disorders and a higher risk of miscarriage and even RM. However, to date, the literature concerning the functions of Breg cells in immune tolerance in pregnancy is limited to only one study, which revealed a beneficial impact in successful delivery 71. In addition, whether the impaired functions of Breg cells in HT are related to adverse pregnancy outcomes requires more extensive research.

### NK cells

The different subtypes of NK cells, including CD16^+^CD56^dim^ (90%), CD16- CD56^bright^, and CD162, play a central role in resisting viruses and inhibiting the early spread of tumors by secreting cytokines, such as IFN-γ, and mediating antibody-dependent cell-mediated cytotoxicity (ADCC) 72. However, some studies demonstrated that the frequency of CD56^dim^ NK cells in the PB of euthyroid women with HT was significantly higher than that in healthy controls 15, 18, 73. This finding indicates that abnormally activated NK cells are possibly involved in the progression of HT and subsequent complications. The current study finds that CD56^bright^ CD25^+^ NK cells, which are regarded as decidual NK (dNK) cells and constitute 70% of decidual immune cells during a normal pregnancy, are considered to play a pivotal role in trophoblast invasion and uterine spiral artery remodeling and exert a regulating effect at the maternal-fetal interface 74. However, instead of dNK cells, the high levels of CD16^+^CD56^dim^ NK cells derived from other body parts through blood circulation can impair maternal tolerance to the fetus. As shown in most recent studies, the percentage of peripheral CD3^-^ CD56^+^ CD16^+^ NK^dim^ cells in women with RM is higher than that in healthy controls (*p*< 0.0001) 75. Thus, altogether, these results imply that the abnormal activation of CD56^dim^ NK cells under HT conditions may share the same cytotoxic effects on the fetus during gestation and lead to early miscarriage in women.

### PD-1

Although programmed cell death-1 (PD-1) is widely identified as an essential target that promotes the invasion and metastasis of tumors 76-78, it also plays an indispensable role in maintaining maternal-fetal tolerance when coexisting with T cell immunoglobulin mucin-3 (Tim-3). For example, in an animal-based experiment 21, the augmented coexpression of PD-1 and Tim-3 on CD4^+^ T cells promoted the predominant production of Th2 cells, and the increased secretion of Th2-type cytokines, such as IL-4 and IL-10, contributed to maintaining normal pregnancy. Although many studies have revealed that PD-1 and programmed cell death-ligand 1 (PD-L1) are highly expressed in HT tissues 34, 48, 79, 80 regardless of whether differentiated thyroid carcinoma (DTC) coexists, it seems to be the response to HT conditions in the immune microenvironment. However, the poor suppressive function of the moderating effect is unable to modify the inflammatory phenomenon in the thyroid and accidentally promotes the metastasis of DTC 80, 81. To date, whether a difference exists in the PD-1 expression level on the maternal-fetal interface between women with HT and non-HT women during early pregnancy, which may have potential clinical applications for predicting the occurrence of miscarriage, still remains inconclusive.

### Tim-3

Regarding Tim-3, which is an important inhibitory molecule, numerous studies have demonstrated its essential role in maintaining early pregnancy 82-84. Tim-3 plays a positive role in the establishment and maintenance of maternal-fetal tolerance by regulating maternal decidual CD8^+^ T (dCD8^+^T) cell responses and the subpopulation of NK cells. In one study 84, a distinct NK cell subpopulation, i.e., Tim-3+ NK cells, was identified to display immunosuppressive activities, including the production of anti-inflammatory cytokines and the induction of Treg cells. However, Tim-3+ NK cells from patients with RM were less capable of inducing forkhead box P3 (Foxp3)^ +^ T cell generation in early pregnancy than cells from normal pregnant women. To the best of our knowledge, only a few research reports have explained the role of Tim-3 in the progression of ATD. However, there were no functional polymorphisms in the Tim-3 gene among the patients with HT and normal subjects in one small sample study 85. Nonetheless, the expression of Tim-3 at the maternal-fetal interface in women with HT, especially women with HT-related miscarriage, is worthy of further exploration because the inhibitory effect of Tim-3 has a profound impact on ADs and systemic immunity. Thus, Tim-3^+^ cells may not only become a biological marker for predicting the occurrence of miscarriage during early pregnancy but also be a potential target in immunotherapy for HT-related miscarriage.

### FoxP3

Generally, Treg cells are involved in maintaining immune homeostasis and self-tolerance by inhibiting the pro-inflammatory activities of CD4^+^ and CD8^+^ effector T cells, NK cells, and antigen-presenting cells. More importantly, FoxP3, which is known as a critical transcription gene for Treg cell function, is regarded as an additional marker of the regulation of maternal immune tolerance 86. However, the expression and polymorphisms of FoxP3 in patients with HT are lower and higher, respectively, than those observed in controls, which are often accompanied by decreased proportions of CD4^+^CD25^+^FoxP3^+^ cells 33, 48, 87, 88. Therefore, the low expression level of Treg cells in women with HT may cause insufficient immunosuppression during pregnancy, which could impair the maternal tolerance to the semiallogeneic fetus and fetal development 89.

Indeed, multiple immune pathways are significantly correlated with HT and can further influence the process of pregnancy (**Table 1**), although the mechanisms by which circulating TPOAb or HT mediates systemic immune disorders are still unclear. In addition, with the development of cellular and molecular immunology, a range of immunotherapies aiming to decrease the miscarriage rate, including TNF-α inhibitors 90, intravenous immunoglobulin (IVIG) 59, 75, and other pathway inhibitors, have been introduced to clinical trials 91-93. However, we need to seriously consider the complications that such treatments may cause in pregnant women. Particularly in women with HT coexisting thyroid carcinoma (TC), suppressing the immune system may promote the central lymph node metastasis (CLNM) of tumors. Furthermore, high-quality studies are needed to reveal the immune-mediated mechanisms because understanding the immune-pathogenic mechanism underlying HT-related miscarriage is pivotal for the development of novel immunotherapies.

## Treatments for HT-related miscarriage

**Figure 4:**
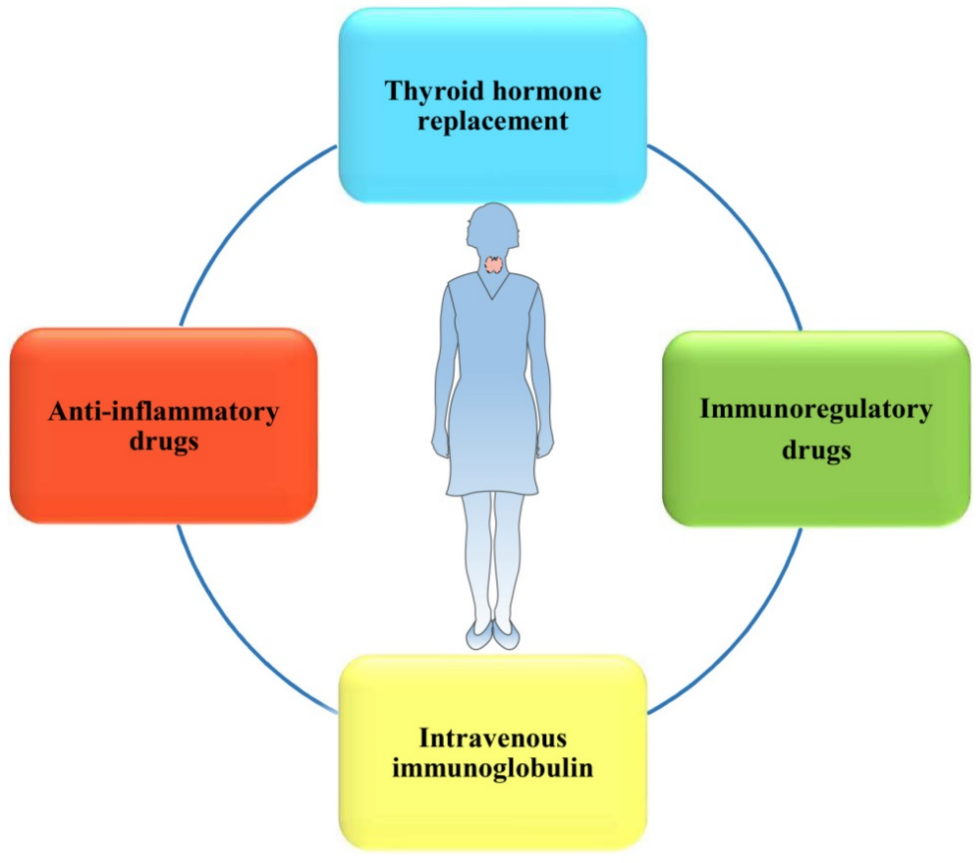
There are multiple treatments for women with HT to lower the risk of miscarriage and even RM, and the efficacies of them remain to be determined.

Currently, multiple treatments for HT-related miscarriage have been widely introduced to clinical trials or practices (**Figure 4**). Indeed, L-T4 supplementation, which is recommended by the American Thyroid Association (ATA) 32, is still the main treatment used to decrease the incidence of overt hypothyroidism during pregnancy among euthyroid women. Immunoregulatory drugs, such as selenium 94, 95 and vitamin D3 96, have been discovered to efficiently alleviate HT progression and regulate the immune system, which may benefit pregnancy outcomes. However, the efficacies of these drug therapies are still controversial in different studies. In addition, to rebalance the immune system, IVIG may become a novel, potentially more effective therapeutic strategy used to maintain pregnancy in some women with HT.

### Levothyroxine

Currently, L-T4 remains the gold standard in the treatment of patients with hypothyroidism 97 and ranks as one of the most widely used drugs worldwide. Since the last century, a certain amount of evidence 23, 98 has demonstrated that adequate L-T4 supplementation (25-50 μg/d as a typical starting dose) before pregnancy can decrease the risk of HT-related miscarriage and premature birth. L-T4 can not only maintain normal thyroid hormone levels but also inhibit the TSH level (below 2.5 mU/L) 32. However, with the exploration of autoimmune thyroiditis, many studies 10, 22, 24, 25 reached the contrary conclusion that regular L-T4 substitution could not decrease the risk of miscarriage in TPOAb-positive women (**Figure 1B**). Even in a recent report 99, a debate existed regarding whether a TPOAb-positive woman with a history of miscarriage needs low-dose L-T4 treatment to increase her chances of conceiving. Notably, one randomized controlled trial (RCT), performed by Dhillon‑Smith et al. 22 concluded that there was no significant difference in the live birth rate between the group of TPOAb-positive euthyroid women who received L-T4 tablets (50 μg/d) for six months and the group of TPOAb-positive euthyroid women who were treated with placebo alone. The authors further indicated that pregnant women with HT cannot benefit from L-T4 supplementation decreasing the risk of adverse pregnancy outcomes. Notably, as these authors discussed, the main limitation of this trial was the fixed-dose of the L-T4 tablet supplementation instead of using a dosage based on the body mass index (BMI), which may be insufficient for women with a high BMI and, thus, could lead to negative results. Therefore, it is reasonable to consider adjusting the dose of L-T4 based on the serum TSH and thyroxine (T4) concentrations measured at each follow-up visit. Most recently, two meta-analyses performed by the group of Rao et al. 23, 98 confirmed that L-T4 supplementation treatment reduced the risk of miscarriage and preterm birth in women with HT with naturally conceived pregnancies. However, in women who underwent *in vitro* fertilization (IVF) or intracytoplasmic sperm injection (ICSI), the efficacies of L-T4 supplementation in preventing miscarriage or preterm birth were insufficient. Therefore, RCTs with larger sample sizes and different races and regions are needed to strengthen the evidence regarding the positive effects of L-T4 in decreasing the miscarriage rate among thyroid autoantibody-positive women.

Furthermore, L-T4 is a critical-dose drug because slight changes in the blood concentration may result in treatment failure and iatrogenic thyrotoxicosis. Therefore, for easier compliance and management, novel thyroxine formulations, including liquid preparations and softgel, have been invented in recent years 100. Compared with traditional L-T4 preparations (tablets), these new types of L-T4 preparations at the same dose perform better in suppressing the serum TSH values and obtaining normal thyroid hormone levels, especially in pregnant women 101, unselected patients without evident malabsorption 102, 103 and even euthyroid patients 104. Choosing a more suitable L-T4 preparation for euthyroid women can not only reduce the impact of the drug absorption rate on the resulting efficacy in decreasing the miscarriage rate but also benefit precision medicine with individualization of the L-T4 dose.

### Selenium

Selenium is an essential trace mineral, and the thyroid has a higher concentration of selenium than most other organs, reflecting the importance of selenium for thyroid metabolism 105. Selenium has been identified as a component of multiple enzymes that have numerous functions ranging from antioxidant and anti-inflammatory roles to the production of active thyroid hormone 106. Selenium deficiency can not only impair thyroid hormone synthesis and metabolism but also lead to an imbalance in the immune system 107. Many surveys have demonstrated that women with HT often have selenium deficiency, which may reflect the potential immune disorders in these patients 94, 108. Meanwhile, numerous studies have shown that a significant decrease in serum anti-thyroid antibodies was present in patients who received Se supplementation. For instance, as determined in a systematic review and meta-analysis, Se supplementation (60-200 μg/d in different trials) can significantly decrease the anti-thyroid antibody level, especially the serum TPOAb level, in the L-T4-treated population 109. This result is consistent with findings showing a decreasing autoantibody titer in another double-blind RCT in Italy (45 cases), 110. The latter study further concluded that Se supplementation was safe during pregnancy and after delivery at a dosage of 83 mcg/d. 110. Interestingly, some research did not find that isolated Se supplementation can prevent the progression of HT 111-113, while combined treatment with L-T4 supplementation was observed to improve the therapeutic effects in delaying the deterioration of chronic inflammation in HT compared with L-T4 monotherapy 114. Thus, the efficacies of Se supplementation in ATDs and even pregnancy outcomes remain controversial. The main reasons for this divergence could be the different doses and preparations of Se (200 μg/d vs. 60 μg/d) used in these trials and whether the individual treatments were combined with L-T4 supplementation. Selenium compounds, such as selenomethionine, sodium selenite, and selenium yeast, which are prescribed to patients, vary among studies 109. Furthermore, there are still many other confounding factors in the inclusion criteria, such as other nutritional deficiencies, geographical location, dietary habits, initial thyroid function, and other concurrent NEADs. Some previous studies have found that women suffering from miscarriage or RM had lower concentrations of Se than healthy controls 115, 116. Even in two recent review reports, both studies further highlighted that Se deficiency is significantly associated with disorders related to human reproduction and pregnancy 117, 118. However, sufficient evidence regarding the efficacy of Se supplementation in decreasing antithyroid antibodies and preventing miscarriage in euthyroid women is lacking 112, 119, 120. Therefore, more studies are needed to provide better clinical evidence proving the ability of Se to reduce thyroid inflammation. Additionally, evidence suggesting that thyroid antibodies can directly damage the placenta is limited. Moreover, a high concentration of selenium in PB could be toxic to pregnant women and their fetuses 121, 122. Thus, following the ethical principle of “First do no harm” and the recommendation in the 2017 ATA guideline 32, currently, high-level evidence supporting the routine use of Se supplementation in TPOAb-positive women during pregnancy to decrease the risk of miscarriage is lacking.

### Vitamin D3

Vitamin D has been found to play an increasingly crucial regulatory role in bone metabolism, the mucosal barrier, and the immune system in the past few decades. More recently, numerous studies have highlighted that an insufficient level of 25-hydroxyvitamin D [25(OH)D] is significantly associated with HT 123-125.

Thus, 25(OH)D supplementation seems to be a potentially effective method for alleviating HT progression and moderating the immune system to achieve a better pregnancy. For instance, one RCT found that women with HT treated with cholecalciferol (vitamin D3) supplementation for three months had a significant decrease in the Th17/Th1 ratio (*p*< 0.046) and enhanced expression of IL-10, even though the serum vitamin D level in the two groups did not significantly differ after the treatment 35. In addition, the efficacy of vitamin D in decreasing the TPOAb level is still debatable. Some studies 126, 127 found that vitamin D3 can significantly reduce the TPOAb levels, but one study 128 reached the following different conclusion: there was no significant reduction in the TPOAb levels in the vitamin D group compared with the placebo group (*p* = 0.08). Indeed, many beneficial effects of vitamin D3 supplementations in patients with HT have been observed in different clinical trials. However, the dose-limiting toxicities of vitamin D3 are also nonnegligible risk factors during treatment. Further studies are needed to obtain better clinical evidence to support clinical decision making, especially for women with HT, and determine whether they can benefit from these immunomodulatory drugs, including Se and vitamin D3, before and during gestation.

### Intravenous immunoglobulin

IVIG has been regarded as a lifesaving treatment for patients with primary immunodeficiency or severe infection in the last centuries 129, 130. More importantly, during the past few decades, accumulating studies have found that IVIG could regulate the immune system, such as NK cells, Th1 cells, and Th2 cells, which might be helpful for patients with ADs 131-133. Many studies have discovered that women with RM presented an abnormal activation of the immune system 134-136; IVIG is currently introduced as a novel treatment for women with RM. Expectedly, the results are encouraging 59, 137-141 and show that IVIG can significantly increase reproductive success in women with a history of RM. For instance, Ahmadi et al. 59 found that IVIG can dramatically regulate the Th1/Th2 ratio by decreasing Th1 (CD4^+^ IFN-γ^+^) cells and increasing Th2 (CD4^+^ IL-4^+^) cells (*p*<0.0001). Additionally, the birth rate in the IVIG group was significantly higher than that in the control group (87.5% vs. 41.6%, *p*<0.0001). Furthermore, in another study 75, the authors demonstrated that IVIG could decrease the number of NK cells and the activating receptors KIR2DS1, KIR2DS4, and NKG2D in women with RM. As some research concluded, low-dose (0.2 g/kg) IVIG therapy can significantly increase the live birth rate in women with HT by rebalancing the functions of the immune system 142, 143.

Nonetheless, in recent years, some scholars 144, 145 have held different views regarding this immunotherapy in women with RM. On the one hand, in some earlier research 146, 147, compared with the control (placebo) group, IVIG had no significant beneficial effect on decreasing the miscarriage rate in women with RM. On the other hand, in contrast to CD56^dim^ NK cells, dNK 148-150 cells perform poorly in exhibiting cytotoxicity or secreting IFN-γ. However, these cells play an essential role in regulating maternal tolerance to the semiallogeneic embryo and the invasion of trophoblast cells, which is essential for forming the placenta. Numerous immunotherapies may harm this process without any benefit. In addition, IVIG could affect the maternal immune system without guaranteed beneficial results and is often accompanied by mild adverse events.

In recent years, different studies have reported that IVIG has positive impacts on increasing the live birth rate in women with RM 59, 75, 142; however, the high cost per infusion and possible side effects, such as fever and even anaphylaxis, should be carefully considered during the treatment decision-making process. Due to the lack of high-level clinical evidence, IVIG treatment is still not recommended for euthyroid women with a history of recurrent pregnancy loss. Further RCTs are needed to determine whether IVIG treatment has long-term effectiveness in women who suffer from HT-related miscarriage and evaluate the comparative efficacy with drug therapies at different levels.

## Conclusion

Although significant progress has been made in our understanding of the contributions of immune dysfunctions triggers to HT related miscarriage, its pathogenesis is still not fully comprehended. In recent years, it confirmed the abnormal expression levels of multiple immune cells and cytokines in HT patients, especially the proinflammatory polarization of naïve T cells and NK cells. Meanwhile, the role of Breg cells in immune-mediated diseases has been also recognized, but the significance of the participation of Breg cells in the progression of HT is rather insufficient. The specific changes of these immune cells and cytokines can not only positively contribute to a better understanding of the role of immune dysfunctions in HT women with adverse pregnancy outcomes, but also provide further insights into the novel treatment for HT related miscarriage. Furthermore, even though multiple immunotherapies in decreasing the risk of HT related miscarriage have been introduced to the clinical trials and practice, the efficacies and safety of these drugs are still needed to have further evaluation. Nevertheless, the rapidly advancing in this field is becoming the most potential treatment component of HT related miscarriage.

## Figures and Tables

**Table 1:** Expression levels of immune cells and cytokines in Hashimoto's thyroiditis

Components	Expression (U/D/I)	Function and correlation with miscarriage and RM	Reference
**Cells**			
Th1	U	Proinflammatory; The low level of Th1 cell expression has a positive effect on placenta formation, but high expression of cytotoxic factors levels, including IFN-γ and TNF-α, will impair immune tolerance at the maternal-fetal interface during pregnancy.	58, 62, 151
Th2	D	Anti-inflammatory; Th2 cells can significantly inhibit the activation of T cells and promote immune tolerance at the maternal-fetal interface.	16, 152
Th1/Th2	U	The ratio of Th1/Th2 reflects the functional balance between proinflammatory and anti-inflammatory cytokines in the human immune system. The higher this ratio, the more severe inflammatory reaction is, which can impair the process of pregnancy.	16, 58, 59
Th17	U	Proinflammatory; The Th17 cells' responses are often overwhelming in HT, which may cause the immune system to shift towards an inflammatory profile and impair the immune tolerance at the maternal-fetal interface during pregnancy.	50, 61
Th22	U	Immunoregulatory; The Th22 cell can regulate the chronic inflammatory reaction, which is crucial for embryo implantation and the success of pregnancy.	51, 61
Tfh	U	Immunoregulatory; Memory Tfh cells have a central role in the regulation of the adaptive immune response. A higher number of Tfh is observed in RM women than controls.	15, 34, 153
Treg	D	Treg cells can inhibit effector immunity, contain inflammation, and support maternal vascular adaptations. Insufficient Treg numbers or inadequate functional competence are implicated in RM	20, 33, 154
Breg	U/I	Anti-inflammatory; Breg can both suppress the pro-inflammatory response, mostly by the production of IL-10 cytokine, and enhance the activity of Treg cells. But dysfunctions of Breg cells were determined in PB of HT patients. Whether it could further impair the immune tolerance at the maternal-fetal interface during pregnancy need more research to evaluate.	65-67, 69, 71
NK	U	Proinflammatory; dNK cells, one of NK cell subsets, are important to placenta formation in early pregnancy. But abnormal activated NK^dim^ cells can cause miscarriage and even RM by cytotoxic activities.	73, 75, 144
**Cytokines**			
IFN-γ	U	Proinflammatory; By increasing the expression of MHC-I and MHC-II and stimulating NK and Th1 inflammatory responses, abnormal IFN-γ responses can induce the miscarriage and RM.	4, 155, 156
TNF-α	U	Proinflammatory; TNF-α, same as TNF-β, has the cytotoxic effects, but whether TNF-α is associated with miscarriage needs more exploration.	157, 158
IL-2	U	Proinflammatory; It promotes the polarization of Th1 cells and immune responses, but whether it can impair the pregnancy need further research.	159, 160
IL-4	D	Anti-inflammatory; IL-4 is one of the important anti-inflammatory cytokines in regulating immune tolerance at the maternal-fetus interface.	55, 56, 58, 63
IL-10	D	Anti-inflammatory; IL-10 is one anti-inflammatory cytokine with important immunoregulatory functions and plays an important role in a successful pregnancy.	54, 66, 161
IL-17	U	Proinflammatory; It was observed increasing in healthy pregnancy but not in spontaneous abortion.	162-165
IL-21	U	Proinflammatory; IL-21 can induce the Th17 differentiation, inhibit the Treg development, and modulation of antibody responses of B lymphocytes. But further research needs to investigate the correlation between IL-21 and miscarriage.	166-168
IL-22	U	Anti-inflammatory; High expression levels of IL-22 potentially represent the reparative processes of organisms, which may be a biomarker of placental dysfunction caused by chronic inflammation.	169-172
IL-23	U	Proinflammatory; It plays an important role in early pregnancy, while the abnormally increased expression level of IL-23 can induce a miscarriage.	173-175
Granzyme B	U	Cytotoxic effect; high levels of Granzyme B may impair the embryonic development.	176
TGF-β	D/I	Anti-inflammatory; The expression level of the TGF-β is related to hormone levels during pregnancy, which is important to maintain pregnancy.	177-180
**Other factors**			
PD-1/PD-L1	U	Anti-inflammatory; High expression levels of PD-1 and PD-L1 in the uterine decidua can inhibit the activity of the inflammatory cells, which are critical for the success of the pregnancy. However, it does not seem that increased expression levels of PD-1 and PD-L1 in HT tissues are beneficial to decrease the risk of miscarriage.	79, 181, 182
FoxP3	D/I	Anti-inflammatory gene; the low expression and splice variants of FoxP3 induce Treg function defect and further impair the expression level of CD4^+^CD25^+^FoxP^+^ cells, which may increase the risk of miscarriage.	33, 88, 89, 183
Tim-3	^†^D	Anti-inflammatory gene; combined with PD-l, Tim-3 signaling can enhance the expression levels of immunosuppressive cells to promote the maternal-fetal immune tolerance.	21, 82-84, 184

^†^D: Lower expression of Tim-3 than controls was observed in Graves' disease patients with thyroid-associated ophthalmopathy but not in HT patients.

## Abbreviations

HT: Hashimoto's thyroiditis
ATD: autoimmune thyroid disease
RM: recurrent miscarriage
Se: selenium
IVIG: intravenous immunoglobulin
CLT: chronic lymphocytic thyroiditis
ADs: autoimmune diseases
NEADs: nonendocrine autoimmune disorders
E2: estradiol
SH: subclinical hypothyroidism
Treg: T regulatory cells
Breg: B regulatory cells
Th2: T helper type 2
L-T4: L-thyroxine
TgAb: thyroid globulin antibody
TPOAb: thyroid peroxidase antibody
TSH: thyroid-stimulating hormone
PB: peripheral blood
IL: interleukin
TGF-β: transforming growth factor-beta
Th1: T-helper type1
Th17: T-helper type17
INF-γ: interferon-gamma
TNF-α: tumor necrosis factor-alpha
EVTs: extravillous trophoblast cells
ADCC: antibody-dependent cell-mediated cytotoxicity
dNK: decidual NK
PD-1: programmed cell death-1
Tim-3: T-cell immunoglobulin mucin-3
PD-L1: programmed cell death-Ligand 1
DTC: differentiated thyroid carcinoma
dCD8^+^T: decidual CD8^+^T
FoxP3: forkhead box P3
TC: thyroid carcinoma
CLNM: central lymph node metastasis
ATA: the American Thyroid Association
RCTs: randomized controlled trials
BMI: body mass index
T4: thyroxine
IVF: vitro fertilization
ICSI: intracytoplasmic sperm injection
25(OH)D: 25-hydroxyvitamin D
Vitamin D3: cholecalciferol
PTC: papillary thyroid carcinoma
PT: partial thyroidectomy
U/D/I: up-regulated/down-regulated/impaired
Tfh: follicular helper T cell
MHC: major histocompatibility complex
